# Increased Expression of *NOTCH-1* and T Helper Cell Transcription Factors in Patients with Acquired Aplastic Anemia

**DOI:** 10.61186/ibj.3754

**Published:** 2022-07-31

**Authors:** Vandana Sharma, Manju Namdeo, Prabin Kumar, Dipendra Kumar Mitra, Parthaprasad Chattopadhyay, Sudha Sazawal, Rekha Chaubey, Renu Saxena, Uma Kanga, Tulika Seth

**Affiliations:** 1 Department of Hematology, All India Institute of Medical Sciences, New Delhi, India;; 2 Department of Transplant Immunology and Immunogenetics, All India Institute of Medical Sciences, New Delhi, India;; 3 Department of Biochemistry, All India Institute of Medical Sciences, New Delhi, India

**Keywords:** Anemia, Aplastic, Transcription factors

## Abstract

**Background:**

Acquired aplastic anemia is an autoimmune disease in which auto-aggressive T cells destroy hematopoietic progenitors. T-cell differentiation is controlled by transcription factors that interact with NOTCH-1, which influences the respective T-cell lineages. Notch signaling also regulates the BM microenvironment. The present study aimed to assess the gene expressions of *NOTCH-1* and T helper cell transcription factors in the acquired aplastic anemia patients.

**Methods:**

Using quantitative real-time PCR, we studied the mRNA expression level for *NOTCH-1*, its ligands (*DLL-1* and *JAG-1*), and T helper cell transcription factors (*T-BET*,* GATA-3*, and *ROR-γt*) in both PB and BM of aAA patients and healthy controls. Further, patients of aplastic anemia were stratified by their disease severity as per the standard criteria.

**Results:**

The mRNA expression level of *NOTCH-1*,* T-BET*, *GATA-3*, and *ROR-γT* genes increased in aAA patients compared to healthy controls. There was no significant difference in the mRNA expression of Notch ligands between patients and controls. The mRNA expression level of the above-mentioned genes was found to be higher in SAA and VSAA than NSAA patients. In addition, *NOTCH-1* and T helper cell-specific transcription factors enhanced in aAA. We also observed a significant correlation between the genes and hematological parameters in patients.

**Conclusion:**

The interaction between *NOTCH-1, T-BET, GATA-3*, and *ROR-γT* might lead to the activation, proliferation, and polarization of T helper cells and subsequent BM destruction. The mRNA expression levels of genes varied with disease severity, which may contribute to pathogenesis of aAA.

## INTRODUCTION

Acquired aplastic anemia is a life-threatening autoimmune disease featured with PB pancytopenia and a hypocellular BM. Over the years, many studies have shown the evidence of immune-mediated mechanisms of BM failure in aAA. It is believed that T-cell subsets and their effector cytokines destroy hematopoietic stem cells in aAA^[^^1^^-^^3^^]^. Th1 (interferon-gamma-producing CD4^+^ T cells), Th17 (IL-17A-producing CD4^+^ T cells), and in some cases Th2 (IL-4-producing CD4^+^ T cells) are expanded in aAA^[^^2^^]^. An effective treatment strategy for aAA is immunosuppressive therapy with anti-thymocyte globulin and cyclosporine^[^^3^^]^.

The Notch pathway controls many cellular functions such as organogenesis and T-cell differentiation^[^^4^^]^. There are four Notch receptors, Notch 1-4, and five ligands, DLL1, 3, 4 and JAG-1 and -2. DLL ligands promote Th1 and Th17 differentiation, while JAG ligands promote Th2 differentiation^[^^5^^,^^6^^]^. Notch exerts its effect via canonical and non-canonical pathways. In the canonical pathway, Notch receptor-ligand binding results in the enzymatic action of γ-secretase complex on intracellular Notch domain. The Notch intracellular domain then binds to the nucleus with CSL to activate the target genes. However, in the non-canonical pathway, Notch acts independent of ligands and CSL interaction. Communication between T helper cell transcription factors (T-BET for Th1, GATA-3 for Th2, and ROR-γT for Th17) and Notch pathway leads to precise lineage determination of CD4^+^ T cells^[^^7^^,^^8^^]^. The Notch pathway is implicated in many autoimmune diseases^[^^9^^,^^10^^]^. Elevated mRNA expression of *NOTCH-2*, *-3*, *-4* and *Hes-1* found in T helper cells from rheumatoid arthritis patients highlights active notch signaling in rheumatoid arthritis^[^^11^^].^ Increased production of NOTCH-1 intracellular protein in T cells has been shown in a mouse model of aAA^[^^12^^].^ It has also been implicated that Notch has a role in the pathogenesis of aAA^[^^13^^,^^14^^]^.

The incidence of aAA has been reported to be higher in India and Asian countries than the western countries; however, there is paucity of molecular data on aAA in the country^[^^15^^]^. In India, a few research groups have studied chromosomal breakage and telomere mutations and reported shorter telomere length in aAA patients^[^^16^^-^^18^^]^. There is still an urgent need to conduct more molecular research in aAA for better understating of the disease pathogenesis and profile of Indian patients. Thus, we aimed to understand the molecular profile of aAA patients, by studying the mRNA expression of *NOTCH-1*, its ligands (*DLL-1 *and* JAG-1*), and T helper cell transcription factors (namely *T-BET*,* GATA-3*, and *ROR-γT*) in both PB and BM of aAA patients and healthy controls. Due to pancytopenia in patients, it was difficult to obtain sufficient samples. Therefore, this study was conducted in a small cohort of patients for generating pilot baseline data.

## MATERIALS AND METHODS

**Patients and controls**

This study recruited 25 untreated aAA patients (6 females and 19 males; mean age 25 years, range 13-60 years), diagnosed according to the Camitta’s criteria^[^^19^^]^, presenting to the Department of Hematology, All India Institute of Medical Sciences (AIIMS), New Delhi. All the patients had normal cytogenetics, and their chromosomal fragility screening test was negative. Prior to the enrollment of the patients, FLAER-based flow cytometry was carried out to diagnose paroxysmal nocturnal hemoglobinuria. The patients were classified into NSAA, SAA, and VSAA, as per their disease severity^[^^20^^,^^21^^]^. The patients belonging to SAA and VSAA categories were also grouped together (SAA + VSAA). The disease severity and hematological characteristics of the patients are listed in Table 1. PB and BM samples from all the patients were collected simultaneously. The control group comprised of 39 individuals (15 females and 24 males; mean age 32 years, range 11-60 years). Healthy PB samples were obtained from 25 healthy volunteers, and normal BM samples were obtained from 14 subjects undergoing nonunion BM grafting at the Orthopedics Department, AIIMS.

**Table 1:** Characteristics of the enrolled aAA patients

**Patient characteristics**	**Value**		**Mean age (y)**	**Gender**
NSAA	18		25	M = 13; F = 5
SAA + VSAA	4 + 3		26	SAA (M = 3; F =1) VSAA (M = 3; F = 0)
Hematological characteristics at diagnosis (mean values with ranges)		NSAA		SAA + VSAA
Hemoglobin (g/L)^*^	61.9 (42-100)			56.2 (44-68)
Platelet count (10^9^/L)	30.4 (12-59)			16.1 (10-29)
Absolute neutrophil count (10^9^/L)	1.14 (0.5-4.7)			0.25 (0.09-0.4)
Retic count (%) (corrected)	0.31 (0.17-0.55)			0.26 (0.15-0.57)

^*^A few patients were transfused; M, male; F, female

**Mononuclear cell isolation from PB and BM**

Mononuclear cells were isolated from EDTA blood (8 ml) and BM (1 ml) samples by Ficoll-Hypaque gradient centrifugation. The cells were washed twice with RPMI-1640 (Sigma-Aldrich, St. Louis, USA) supplemented with fetal calf serum and used for RNA isolation

**RNA isolation and quantitative real-time PCR**

Total RNA was isolated from PB and BM mononuclear cells using TRI reagents (Sigma-Aldrich) according to the manufacturer's instruction. Complementary DNA was synthesized with RevertAid RT enzyme (Thermo Fisher Scientific, Massachusetts, USA) using random primers. Quantitative real-time PCR for *NOTCH-1*,* DLL-1*,* JAG-1*, *T-BET*,* GATA-3*,* ROR-γT*, and *GAPDH *was performed using SYTO-9 fluorescent dye (Thermo Fisher Scientific) in a 7500 Real-Time PCR System (Applied Biosystems, Harvard, USA). The PCR reactions were cycled 40 times after an initial denaturation at 94 °C for 5 minutes, followed by denaturation at 94 °C for 30 seconds, primer annealing at 60 °C for 30 seconds, extension at 72 °C for 30 seconds, and final extension at 72 °C for 10 minutes. *GAPDH* was kept as housekeeping gene for normalizing the expression of each gene using 2^-ΔCt^ method. Fold change in gene expression was also calculated. The fold change in gene expression in aAA patients was compared with controls and categorized as upregulated (two-folds), downregulated (0.5-fold in), and unchanged (fold change in between 0.5 and 2) in comparison with normal controls. Previously published primer sequences for *NOTCH-1*,* JAG-1*,* DLL-1*,* T-BET*, *GATA-3*,* ROR-γT*, and *GAPDH* genes were used^[^^22^^-^^28^^]^.

**Statistical analysis**

Statistical analysis was performed with Graph pad Prism version 5.0. Mann-Whitney U test was used to compare the data. Pearson’s correlation coefficient was also applied for drawing correlations. All the values were expressed as mean ± SD. The p < 0.05 was considered as statistically significant.

## RESULTS

**Relative mRNA expressions in aAA patients vs. controls**

**Fig. 1:**
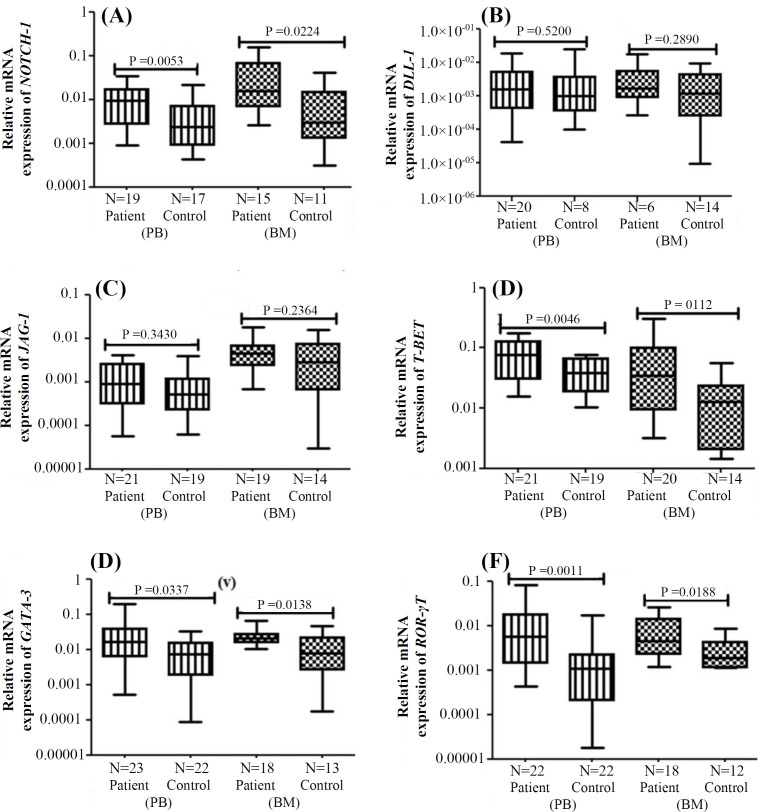
Box and whisker plots representing the relative mRNA expression of (A) *NOTCH-1*, (B) *DLL-1*, (C) *JAG-1*, (D) *T-BET*, (E) *GATA-3*, and (F) *ROR-γT* in PB and BM of aAA patients vs. healthy controls. N, the number of patients/controls

The relative mRNA expression of *NOTCH-1*,* T-BET*,* GATA-3* and *ROR-γT* was significantly elevated both in PB and BM of aAA patients compared to healthy controls. However, there was no significant difference in the relative mRNA expression of Notch ligands, *DLL-1* and *JAG-1*, between the patients and healthy controls in terms of PB and BM (Table 2 and Fig. 1). Similar results were obtained while calculating the fold change of gene expression in aAA patients and controls.

**Relative mRNA gene expression in** **SAA + VSAA vs. NSAA patients**

**Fig. 2:**
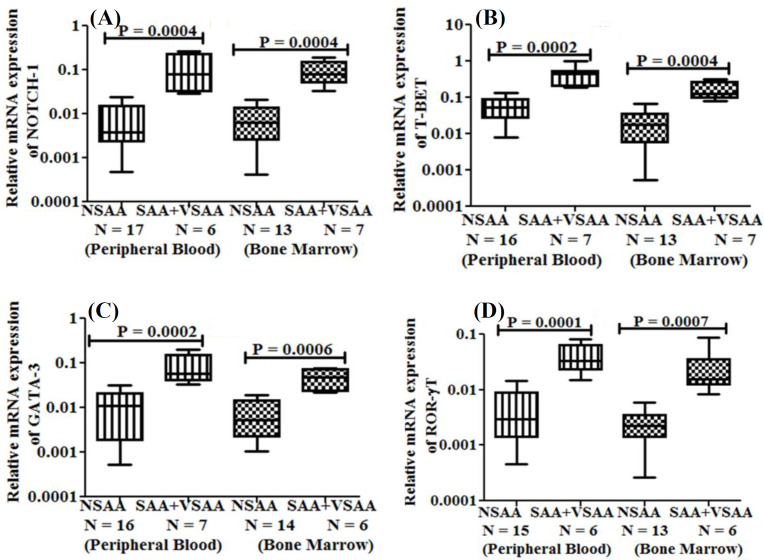
Box and whisker plots representing the relative mRNA expression of (A) *NOTCH-1*, (B) *T-BET*, (C) *GATA-3*, and (D) *ROR-γT* in PB and BM of NSAA vs. SAA + VSAA patients. N, the number of patients; R, correlation co-efficient; P, p value

 Among the different groups of aAA patients in terms of severity, the SAA + VSAA group showed relatively higher mRNA expression of *NOTCH-1*,* T-BET*,* GATA-3*, and *ROR-γT* in PB than NSAA group (Table 3 and Fig. 2). 

**Correlations among relative mRNA gene expression in aAA patients**

**Fig. 3:**
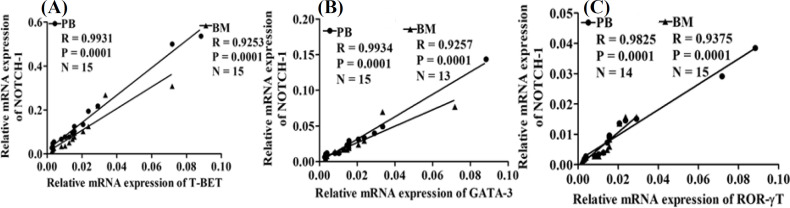
Correlation analysis between relative mRNA expression of *NOTCH-1* and (A) *T-BET*, (B) *GATA-3*, and (C) *ROR-γT* in aAA patients. R, correlation co-efficient; N, number of patients

Significant positive correlations (p < 0.05) were observed among the relative mRNA expressions of *NOTCH-1* and *T-BET, NOTCH-1* and *GATA-3*, and *NOTCH-1* and *ROR-γT* in PB and BM of aAA patients, respectively. The positive correlations depicted a strong association between the above-mentioned genes (Fig. 3). Thus, an increase in the expression of *NOTCH-1* would also elevate the expression of other genes (*T-BET, GATA-3,* and *ROR-γT*).

**Correlations between the relative mRNA expressions and hematological parameters in aAA patients**

Negative correlations were found between hematological parameters (hemoglobin, platelet count, absolute neutrophil count, and reticulocytes) and the relative mRNA expressions of *NOTCH-1, T-BET*,* GATA-3*, and *ROR-γT* genes in PB and BM of aAA 

**Table 2:** Relative mRNA gene expression in aAA patients versus healthy controls

**Relative mRNA expression**	**PB**			**BM**		
	**aAA patients **	**Controls**	**p value**	**aAA patients **	**Controls **	**p value**
*NOTCH-1*	0.01 ± 0.02	0.005 ± 0.006	0.0053	0.04 ± 0.033	0.01 ± 0.01	0.0224
*DLL-1*	0.004 ± 0.005	0.003 ± 0.06	0.5200	0.003 ± 0.004	0.002 ± 0.002	0.2890
*JAG-1*	0.004 ± 0.001	0.003 ± 0.002	0.3430	0.005 ± 0.004	0.004 ± 0.005	0.2364
*T-BET*	0.08 ± 0.05	0.04 ± 0.02	0.0046	0.07 ± 0.09	0.01 ± 0.019	0.0112
*GATA-3*	0.03 ± 0.02	0.01 ± 0.01	0.0337	0.03 ± 0.02	0.01 ± 0.01	0.0138
*ROR-γT*	0.01 ± 0.02	0.002 ± 0.003	0.0011	0.007 ± 0.007	0.003 ± 0.003	0.0188

Numbers are represented as mean ± SD.

**Table 3:** Relative mRNA gene expression in SAA + VSAA vs. NSAA patients

**Relative mRNA expression**	**PB**			**BM**		
	**SAA + VSAA patients**	**NSAA** **patients**	**p ** **value**	**SAA + VSAA patients**	**NSAA** **patients**	**p ** **value**
*NOTCH-1*	0.12 ± 0.10	0.008 ± 0.007	0.0004	0.09 ± 0.06	0.007 ± 0.006	0.0004
*T-BET*	0.4 ± 0.3	0.06 ± 0.04	0.0002	0.02 ± 0.1	0.02 ± 0.02	0.0004
*GATA-3*	0.09 ± 0.07	0.01 ± 0.01	0.0002	0.05 ± 0.03	0.007 ± 0.006	0.0006
*ROR-γT*	0.04 ± 0.02	0.005 ± 0.005	0.0001	0.03 ± 0.03	0.003 ± 0.002	0.0007

**Fig. 4:**
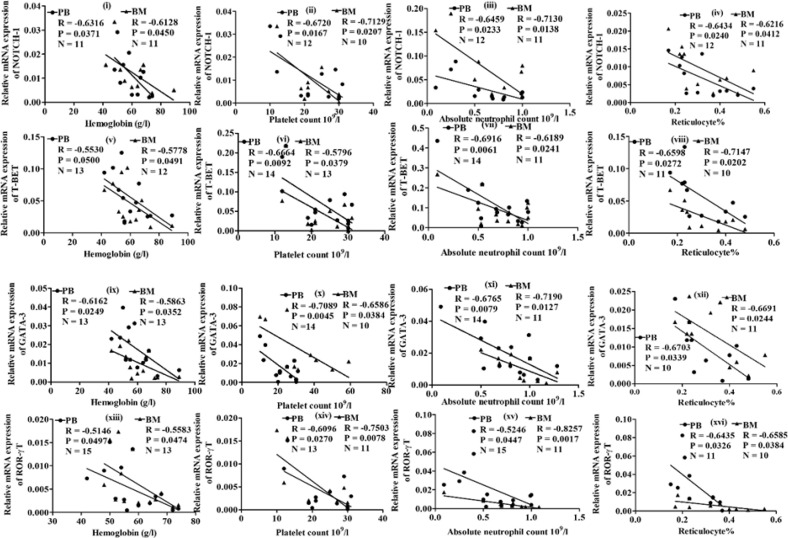
Correlation analysis between the relative mRNA expression of *NOTCH-1*, *T-BET*, *GATA-3*, and *ROR-γT* with hematological parameters of patients. N, number of patients; R, correlation co-efficient; P, p value

Numbers are represented as mean ± SD. All p values were less than 0.05.

patients. A perfect negative correlation has a coefficient of -0.1. R value (correlation coefficient) evaluates the degree of linear relationship between two variables. In our study, the coefficient of correlation (R) ranged from -0.5146 to -0.8257, with p values of less than 0.05, indicating significant strong negative correlations between hematological parameters and the studied genes (Fig. 4). Therefore, the increased expression of *NOTCH-1*,* T-BET*,* GATA-3*, and *ROR-γt* genes may be associated with low level of hemoglobin, platelets, absolute neutrophil count and reticulocytes in patients with aAA. 

## DISCUSSION

In this study, we observed the increased relative mRNA level of *NOTCH-1*, T helper cell transcription factors (*T-BET, GATA-3,* and *ROR-γT*) in both PB and BM of aAA patients, as compared to healthy controls. There was no significant difference in the mRNA level of Notch ligands, i.e. *DLL-1* and *JAG-1*, between aAA patients and healthy controls. Additionally, we did not find any difference in the mRNA expression of the above genes between PB and BM of the patients. Hence, the expression of *NOTCH-1*,* T-BET*,* GATA-3*, and *ROR-γT* genes can be evaluated in the PB of the patients alone, in situations where BM sample is difficult to acquire.

In the present study, PBMNCs were used instead of sorted CD4^+^ cells. Since the aAA patients have trilineage pancytopenia, the limited cells are available, hence, cell sorting would lead to further cell loss. Elevated mRNA expression of *NOTCH-1* in PB and its target genes in aAA patients were reported by other research groups working on aAA PBMNCs^[^^12^^,^^14^^]^. Similarly, in our study we found an enhanced mRNA expression of *NOTCH-1* in aAA patients compared to healthy controls, both in PB and BM. In certain studies, transfusion of aAA PBMNCs with mesenchymal stem cells has led to the increased *Notch-1*,* DLL-1*, and *JAG-1* mRNA level along with the increased number of regulatory T cells and decreased number of Th17 cells. This observation implicates that Notch signaling modulates T helper cells in aAA^[^^14^^,^^29^^]^. 

In this study, we also observed no difference in the mRNA level of *DLL-1* and *JAG-1* ligands between the patients and controls. However, some researchers have reported equivocal results on the mRNA level of *DLL-1* and *JAG-1* in aAA^[^^13^^,^^14^^,^^30^^]^. Similar to our observation, Sodsai and Ma et al.^[^^31^^,^^32^^] ^ found no difference in *DLL-1* and *JAG-1* mRNA expression in various autoimmune diseases. Notch signaling can act non-canonically in the absence of ligands^[^^7^^]^. The variable findings obtained across different studies indicate difference in patient profiles, presence of infections, concomitant therapy or effect of blood transfusions^[^^33^^]^.

We found an increased mRNA level of *T-BET*, which has been also reported by other researchers^[^^14^^,^^34^^-^^36^^]^. There are discrepant reports regarding *GATA-3* and *ROR-γT* mRNA expression in aAA. Some reports have disclosed lower or no difference^[^^37^^-^^40^^]^ in *GATA-3* and *ROR-γT* mRNA expression, and some have demonstrated enhanced level of *GATA-3* and *ROR-γT* gene expression in aAA^[^^14^^,^^24^^,^^41^^]^. Our results are consistent with the studies showing an enhanced mRNA expression level of *T-BET*, *GATA-3,* and *ROR-γT* in aAA. Thus, overexpression of these three genes might lead to hematopoietic abnormality in aAA.

Furthermore, we found an increased mRNA level of * NOTCH-1*, *T-BET*,* GATA-3*, and *ROR-γT *in both PB and BM of SAA + VSAA patients compared to NSAA ones. In this agreement, another study reported an increased mRNA expression of *T-BET* and *ROR-γT* in SAA patients, when compared to NSAA patients^[^^24^^]^. These results suggest the probable influence of aberrant gene expression on disease severity in aAA patients.

Our study depicted positive correlations among *NOTCH-1*,* T-BET*,* GATA-3*, and *ROR-γT* mRNA expressions in aAA patients. *T-BET* and Th1 cytokine IFN-γ are direct targets of *NOTCH-1*. In tandem with *GATA-3* and *ROR-γT*, *NOTCH-1* can regulate Th2 and Th17 cell differentiation, respectively^[^^42^^,^^43^^]^. In addition, *NOTCH-1* constitutively increases its own expression after activation^[^^44^^]^ and subsequently modulates downstream interactions with T helper cell transcription factors.

Negative correlations were observed between the mRNA expressions of *NOTCH-1*,* T-BET*, *GATA-3*, *ROR-γT* and hematological parameters of patients, including hemoglobin, platelet count, absolute neutrophil count, and reticulocytes. The Notch pathway regulates cell proliferation, differentiation, cell death^[^^4^^]^ and interacts with T helper cell transcription factors. Significant correlations between mRNA levels of genes and hematological parameters may have an important association with the bone marrow failure and disease development in aAA. Further studies might assist in development of potential biomarkers for disease prognosis and refine our understanding of the pathophysiology of aAA. Notch signaling can be inhibited with gamma secretase inhibitors^[^^30^^]^. Our study might be utilized to administer gamma secretase inhibitors in aAA patients who have high levels of *NOTCH-1*. 

The findings of the present study showed that the altered mRNA expression of *NOTCH-1* and T helper cell-specific transcription factors, i.e. *T-BET*,* GATA-3*, and *ROR-γT*, are important dynamic molecular mechanisms active in aAA. As their mRNA expressions fluctuate with disease severity, aAA patients with higher *NOTCH *levels may be given alternative therapy with gamma secretase inhibitors. Furthermore, Notch pathway might be active in a non-ligand dependent manner in aAA, as we could not find any difference in *DLL-1* and *JAG-1* mRNA expressions between the aAA patients and control ones. Significant correlations between *NOTCH-1*, transcription factor genes, and hematological parameters highlight the importance of *NOTCH-1* in regulating the developmental cell-fate decisions in aAA. Additional investigations on both canonical and non-canonical Notch pathways, the existing molecular mechanisms, and interactions among the above-mentioned genes provide new insights into their role in pathogenesis of aAA.

## DECLARATIONS

### Acknowledgments

We are grateful to All India Institute of Medical Sciences (AIIMS), New Delhi, India for the financial support, infrastructure, and laboratory facilities.

### Ethical statement

The protocol of this study was conducted in accordance with Helsinki Declaration and approved by the Institutional Ethics Committee of All India Institute of Medical Sciences, New Delhi, India (Ethical code: IEC/T-353/30/08/13). Signed informed consent was obtained from all the patients (or legal guardians, if minors)/controls.

### Data availability

The data supporting the findings of this study are available from the corresponding author upon reasonable request.

### Author contributions

VS: collected the samples, performed the research, contributed in acquisition and analysis, and helped in writing of manuscript; MN: contributed in acquisition and analysis and helped in writing of manuscript; PK: contributed in acquisition and analysis and helped in writing of manuscript; DKM: designed the research study and helped in interpretation of data; PC: designed the research study and helped in interpretation of data; SS: helped in writing of manuscript, critically revised the manuscript, and helped in revision of manuscript; RC, RS, and UK: critically revised the manuscript; TS: designed the research study, helped in interpretation of data, and helped in revision of manuscript. All authors have read and approved the final version of manuscript.

### Conflict of interest

None declared.

### Funding/support

We express our sincere thanks to All India Institute of Medical Sciences (AIIMS; New Delhi, India) for funding this study—AIIMS intramural research grant, A-359.
